# Medical Nutrition Therapy in Critically Ill Patients Treated on Intensive and Intermediate Care Units: A Literature Review

**DOI:** 10.3390/jcm8091395

**Published:** 2019-09-06

**Authors:** Andrea Kopp Lugli, Aude de Watteville, Alexa Hollinger, Nicole Goetz, Claudia Heidegger

**Affiliations:** 1 Intensive Care Unit and Intermediate Care Unit, Intensive Care Medicine and Department of Anesthesiology, University Hospital Basel, 4031 Basel, Switzerland; 2 Division of Intensive Care, Department of Acute Medicine, Geneva University Hospitals, 1205 Geneva, Switzerland; 3 Intensive Care Unit and Intermediate Care Unit, Intensive Care Medicine and Department of Anesthesiology, University Hospital Basel, 4031 Basel, Switzerland; 4 Endocrinology, Diabetes and Metabolism, University Hospital Basel, 4031 Basel & Department of Anesthesiology, University Hospital Basel, 4031 Basel, Switzerland

**Keywords:** nutritional support, medical nutrition therapy, intensive care unit, intermediate care unit, critically ill patients

## Abstract

Medical nutrition therapy in critically ill patients remains challenging, not only because of the pronounced stress response with a higher risk for complications, but also due to their heterogeneity evolving from different phases of illness. The present review aims to address current knowledge and guidelines in order to summarize how they can be best implemented into daily clinical practice. Further studies are urgently needed to answer such important questions as best timing, route, dose, and composition of medical nutrition therapy for critically ill patients and to determine how to assess and to adapt to patients’ individual needs.

## 1. Introduction

The critical importance, complexity, and challenge of clinical nutrition for critically ill patients is best understood when recapitulating the evolution of this complex patient group and, consequently, the proposed guidelines and clinical practice strategies during the last decades, as follows: (1) Adult critically ill patients present as a heterogeneous group with regard to diagnosis, severity of illness, and the number of (pre-)existing comorbidities. (2) Critically ill patients are treated not only on intensive care units (ICU), but increasingly in smooth transition on so-called step-down or intermediate care units (IMC-U) depending on the severity of illness, the available facilities, and hospital internal regulations. Still, these patients should not be systematically divided into separate categories. From a metabolic point of view, all patients requiring intensive or intermediate care suffer from a relevant catabolic stress response, which parallels the severity of injury or illness. From an institutional point of view, IMC-Us are relatively new facilities treating patients who require less care than standard intensive care, but more than that which is available from ward care [1]. Taking these two points into account, an overlap of IMC-U and ICU treatment is owed to the organization and available facilities of each individual hospital, as follows: Patients with the same severity of illness may be treated in one hospital on the ICU and in another hospital on the IMC-U. Since IMC-Us are an emerging type of treatment facility, IMC-U specific randomized clinical trials (RCTs) and guidelines are still missing and should be a field of future research. Therefore, patients in both of these groups are considered to be critically ill patients throughout this review article as their nutritional needs, first and foremost, parallel the course of illness and are not defined by the treating facility per se. (3) The ICU population suffers from a high prevalence of malnutrition when admitted to hospital (up to 60%) and during the intensive care treatment phase [2,3,4,5]. (4) The diagnosis of malnutrition and the assessment of nutritional needs are complex and ask for systematic re-evaluation and adaptation. (5) Some study data from recent years have highlighted the association between protein and caloric deficiency and higher morbidity and mortality rates [2,6,7,8,9]. These findings have introduced the conversion from “nutritional support” as an adjunctive care to “nutrition therapy”, which not only covers micro- and macronutrient needs, but also intends to blunt the metabolic response, cellular injury, and immunological alteration. However, several further studies showed no clinical benefit [10,11,12,13] for higher protein and calorie administration in the ICU or reported harmful results, such as increased adjusted mortality at 6 months for mechanically ventilated patients [10] and less gastrointestinal tolerance in patients with acute lung injury [13]. Additionally, the idea of pharmaco-nutrition was abandoned after several neutral and one lethal trial [14,15,16]. On these grounds, the current European Society for Clinical Nutrition and Metabolism (ESPEN) guideline defines the term “medical nutrition therapy” (MNT), which encompasses oral nutritional supplements, enteral, and parenteral nutrition. Furthermore, ESPEN underlines the need for well conducted trials on optimal protein intake since currently available studies are not comparable in terms of patient selection and calorie and protein intake, as well as timing and route of administration. Yet, ESPEN evokes the possibility that it is possible that, similar to caloric targets, optimal protein targets change over time in the ICU and that a high protein intake is only beneficial if not associated with overfeeding [17]. However, so far this hypothesis is not yet confirmed by RCTs.

To date, nutritional practices are widely diverse [18] and many studies, reviews, and guidelines address only one or some of the relevant aspects of MNT [7,17,19,20,21,22,23,24,25,26,27,28,29,30,31,32,33,34,35,36,37,38,39,40,41,42,43,44,45,46,47]. The ESPEN guidelines [17] and those of the American Society on Parenteral and Enteral Nutrition (ASPEN) [27], as well as the Canadian Critical Care Practice Guidelines (CCPG) [48], are among the most regularly updated evidence-based guidelines. All three underline the relevance of the points stated above and the need for further carefully planned randomized trials. The present review aims to address current knowledge and guidelines in order to summarize how they can best be implemented into daily clinical practice. We specified the different guidelines’ recommendation grades. The ESPEN guidelines use standard operation procedure (grade A, B, 0, or good practice point (GPP)) [49] and the ASPEN guidelines grade the study evidence level, from high to very low for RCTs and low to very low for observational studies; the good practice statement is ungraded [50].

## 2. Screening

A high prevalence of malnutrition is observed at ICU admission [5], with a further energy and protein deficit (50% and 60%, respectively) occurring during ICU stay [51,52]. Indeed, an international multicenter observational study revealed an association between the increase of energy and protein intake and the decrease of mortality in patients with a body mass index (BMI) <25 or >35 kg/m^2^ [6]. The same research group failed to validate this concept in the TOP-UP trial [53]. More generally, no nutritional intervention reduced mortality in critically ill patients, except withholding early glutamine in patients with multiorgan failure [14].

On the other hand, some recent RCTs investigating the timing of initiation as well as the quantity and the route of MNT showed contradictory results [7,54,55,56,57]. Waiting for more conclusive studies, actual guidelines suggest that all critically ill patients should be routinely screened with the aim of preventing malnutrition [17,27].

Many different screening tools to identify the malnutrition risk are available. Some scores are widely used, such as (1) the Nutrition Risk Screening (NRS) 2002 [58], focusing on nutritional state, illness severity, and age; (2) the Nutrition Risk in the Critically Ill (NUTRIC) score [59], including a severity score, comorbidities, and the number of hospital days; (3) the Subjective Global Assessment (SGA), combining historical data and a clinical examination [60]; and (4) the Mini-Nutrition Assessment (MNA) [61], which is specific for the elderly population. Despite the large number of existing scores, none has been validated for the critically ill thus far [62] and none have been able to identify patients who improve with enhanced feeding.

Laboratory data, such as albumin and pre-albumin, are frequently used as markers of nutritional status, but cannot be clearly interpreted during critical illness, as low albumin and prealbumin levels occur as a response to increased inflammation [63].

Weight changes are also difficult to interpret considering the relevant amounts of fluid administration and depletion during the ICU and IMC-U stay. Thus, weight loss does not reflect loss of lean body mass. Nevertheless, this aspect is an important issue for the detection of malnutrition, as the loss of lean body mass has been associated with longer hospitalization and reduced quality of life and functional abilities [60]. Some methods, such as ultrasound [64] or computerized tomography (CT) scan [65], are promising tools that can help to determine the extent of lean body mass loss. However, these methods still need to be validated in clinical practice. Moreover, the way in which they influence therapy is not yet clear. Bioelectrical impedance to assess body composition and lean body mass may be useful, but its interpretation is complicated by important fluid changes that occur in the critically ill [66].

Concerning these screening tools, ASPEN [27] and ESPEN [17] have taken different positions. While the current ASPEN guidelines recommend using either the NRS 2002 or the NUTRIC-Score for nutritional risk determination [27], the new ESPEN guidelines propose a more general clinical assessment including anamnesis, evaluation of muscle mass and strength, unintentional weight loss, and body composition (GPP). Furthermore, all critically ill patients with a length of stay of more than 48 h should be considered at risk for malnutrition (GPP) [17].

## 3. Assessment of Medical Nutrition Therapy

### 3.1. Energy Needs

It is extremely challenging to assess energy requirements of the critically ill, as the requirements depend on many factors and may be dynamic over time [67].

Initially, critical illness provokes an acute early phase [17] characterized by a major metabolic instability with hypermetabolism, insulin resistance, glycemia perturbation, and increased catabolism with the production of endogenous substrates [68]. This is followed by an acute late period with a certain degree of stabilization but persistent catabolism. Finally, when the inflammatory state and critical conditions decrease, the late phase or chronic phase sets in, with the beginning of anabolism and rehabilitation [43]. Moreover, energy expenditure (EE) is also modulated by many other conditions such as underlying illness, inflammatory state, medications, body composition, and nutritional status prior to admission [67].

While predictive formulas to estimate EE are widely used for MNT, many studies have shown them to be inaccurate and imprecise [69,70,71,72]. Up to now, no predictive equation has been validated for the critically ill [17,27]. If predictive formulas are used, hypocaloric MNT (<70% of the estimated EE) should be preferred over isocaloric nutrition during the first week of the ICU and IMC-U stay in order to avoid overnutrition [17].

For some time, the ESPEN guidelines have recommended the use of indirect calorimetry (IC) to determine EE as the best standard practice [73,74]. This attitude is reiterated in the current ASPEN (grade very low) and ESPEN guidelines (grade B) [17,27]. Furthermore, the ESPEN guidelines suggest that if IC is used, an isocaloric nutrition can be progressively implemented after the early phase of acute illness (grade 0). However, during the early phase of acute illness, hypocaloric nutrition (not exceeding 70% of EE) should be administered (grade B). Still, the exact way to use IC measurements to guide MNT needs to be validated by further studies.

The IC measurement is based on O_2_ and CO_2_ concentrations as well as the volume of expired gas/minute [67]. The EE is then calculated directly by the calorimeter according to the Weir’s equation [75]. This measurement can be performed either in mechanically ventilated or spontaneously breathing patients. Measurements are influenced by several factors such as medications, stress, body temperature, mechanical ventilation, agitation, etc. [76,77]. The currently available IC devices are expensive, time consuming, and not always accurate; thus, IC measurements cannot be performed regularly. The International Multicentric Study Group for Indirect Calorimetry (ICALIC) recently developed a new IC aimed at meeting clinical needs [67].

The ICALIC study group suggests IC measurements on day 3 to 4 after admission [67], which should be repeated as soon as clinical conditions change. They suggest adapting energy targets to the result of the last IC measurement with the aim of optimizing nutritional management [67].

If IC is not available, energy expenditure calculated from the carbon dioxide production (VCO2) extracted from the ventilator or oxygen consumption (VO2) from pulmonary arterial catheter are recommended by the new ESPEN guidelines [17]. However, these methods were not shown to be accurate compared to IC measurements [78,79,80] and can only be obtained with invasive monitoring.

### 3.2. Protein Needs

Although the coverage of energy needs is an area that has been studied for several years, the importance of the protein needs has only recently become a subject of focus. Indeed, critical illness causes protein catabolism resulting in important muscle loss, which influences a patient’s survival and clinical outcome [81]. Current recommendations on protein intake for critically ill patients are based mainly on observational data and only a few RCTs having different designs. Thus, it is difficult to draw definitive conclusions.

Various recent observational studies have focused on the association between protein intake and patients’ clinical outcomes, suggesting an association between mortality reduction and protein intake [82,83,84]. For instance, decreased mortality has been related to protein intake ≥1.2 g/kg/day in non-septic and non-overfeed critically ill patients [84] by achieving ≥80% of the prescribed protein intake [82] and to a protein intake of >1 g/kg/day [83].

Conversely, recent RCTs on protein needs have shown contradictory results and up-to-date no mortality benefit with higher amino acid intake could be shown by RCTs. In the Ferrie study (*n* = 120), the administration of 1.2 g/kg parenteral amino acids (AA) compared to 0.8 g/kg was associated with attenuated muscle wasting, less fatigue, and better nitrogen balance, but no difference in mortality or length of stay was observed [85].

The Nephro-Protective trial (*n* = 474), with higher AA administration in the intervention group, did not affect the duration of renal dysfunction but only showed an improved estimated glomerular filtration rate (eGFR) and daily urinary output. Furthermore, serum urea was significantly higher, with a trend toward increased renal replacement therapy (RTT) in patients receiving AA therapy, which was not present after controlling for baseline imbalance in terms of renal failure at the time of study enrollment [86].

Other recent RCTs, such as the EAT-ICU (*n* = 203) and the TOP-UP trial (*n* = 125) on protein needs, have confirmed no differences on clinical outcomes including mortality, hospital stay, days of ventilation, nosocomial infections, or organ failure rates [53,56]. However, the EAT-ICU trial also showed a prolonged ICU length of stay (post-hoc analysis) as well as a trend to increased plasma urea levels in the Early Goal-directed Nutrition group [56].

The EPaNIC trial (*n* = 4640) showed that early initiation of parenteral nutrition (PN) was associated with a longer course of RTT and a longer ICU stay. Similar functional status was observed in the early and late PN group [54]. A prospectively planned subanalysis of the EPaNIC trial found an increased incidence of ICU-acquired muscle weakness with slower recovery in the early-PN group [87]. Apart from this, in another post hoc analysis of the EPaNIC trial, delayed recovery was also attributed to the early administration of amino acids by PN [88].

The results of the recent RCTs underline the urgent need for further studies to provide answers to questions still outstanding, such as the right amount of protein to administer and the best timing.

Based on the available results and without the possibility to assess individual protein needs during a protein-loss phase, ESPEN guidelines [17] suggest a progressive provision of 1.3 g/kg/day of protein equivalent during critical illness (grade 0). In contrast, the ASPEN guidelines [27] propose a protein intake of 1.2–2.0 g/kg/day (grade very low).

Exercise in combination with achieving protein targets has also been suggested to maximally maintain muscle mass [17,89,90].

## 4. Practical Implementation

### 4.1. Special Risk Groups

A few distinct patient subgroups, consisting of critically ill surgical and medical patients at particular risk for malnutrition, are defined and discussed below.

#### 4.1.1. Obese and Bariatric Patients

One steadily growing sub-population consists of patients who are already metabolically challenged (i.e., diabetic, severely obese, and post-bariatric/obese surgery patients) [91].

As the number of bariatric operations increases, one must keep in mind that approximately 30% of bariatric patients develop nutritional complications [91]. Furthermore, the proportion of obese critically ill patients is growing parallel to the increasing prevalence in the general population [92]. The ASPEN guidelines suggest to additionally focus the nutritional assessment of these patients on biomarkers of metabolic syndrome, evaluation of comorbidities, and the level of inflammation [27].

In order to estimate patient nutritional needs, resting EE is best assessed by IC [20]. If not available, energy intake can be guided by adjusted body weight (BW) and protein delivery by urinary nitrogen losses or lean body mass determination (using CT or other tools), according to the ESPEN guidelines [17].

Several existing predictive scores, such as the Mifflin-St Jeor (MSJ) and the Harris–Benedict equations [93,94], can be applied if IC is not available in the case of complicated bariatric patients [20,94]. However, it is important to consider that these scores are not specifically validated for post-bariatric patients and IC is recommended in the first place [17].

The ESPEN guidelines recommend an isocaloric high protein diet including a protein intake of 1.3 g/kg adjusted BW/day (GPP) [17]. On the other hand, the ASPEN guidelines [27] suggest a hypocaloric high-protein feeding protocol, with 65%–70% of measured energy requirements using IC or weight-based equations (calories: BMI 30–50 kg/m^2^ = 11–14 kcal/kg actual BW/day, BMI > 50 kg/m^2^ = 22–25 kcal/kg ideal BW/day; protein: BMI 30–40 kg/m^2^ = 2 g/kg ideal BW/day, BMI ≥ 40 kg/m^2^ = 2.5/kg ideal BW/day), for all classes of obesity.

In order to avoid micronutrient (iron, calcium, vitamin D, vitamin B_12_, folate) deficiency after bariatric surgery, life-long multivitamin supplementation is necessary for post-bariatric patients. Since many patients show malabsorption for iron, a documented deficiency is best supplemented intravenously [91].

#### 4.1.2. Geriatric Patients

Patients aged ≥65 years comprise a large portion of the critically ill population and often struggle with nutritional intake and risk of malnutrition. In the ICU and IMC-U setting, additional acute illness on top of an already frail state often leads to poor outcomes [95]. The assessment of the frailty score in this subgroup might be a valuable addition to malnutrition screening [21].

A state of sarcopenia in acutely ill elderly patients is associated with increased short-term mortality [22]. The course of elderly patients according to their nutritional status (BMI and albumin levels) was evaluated and resulted in a higher prognostic value than their absolute age [23]. However, a recent review emphasized the importance of additional parameters such as energy intake, weight loss, and grip strength, whereas biochemical markers were susceptible to the underlying disease process and were not always reliable [96].

Swallowing and chewing problems may be an important issue affecting oral intake in geriatric patients. Therefore, smaller and more frequent meals completed by oral nutritional supplements (ONS) are suggested [96]. Evaluation of swallowing, dental status, oral health, and potential drug side effects that might impair oral feeding should be conducted systematically [95].

About 20% of older critically ill patients have sarcopenia before hospitalization [22,97] and are subjected to various antianabolic and procatabolic factors during critical illness. An ESPEN expert working group recommends 1.2–1.5 g protein/kg/day in elderly patients suffering or at risk for malnutrition, since they have acute or chronic illness. Even higher protein intakes are recommended for this patient population in case of severe illness or injury [98]. In a recent review of older ICU patients, besides the provision of at least 1.2–1.5 g protein/kg usual BW/day, a combination including regular and early ambulation (if possible) and/or physical therapy and follow-up rehabilitation are prudently recommended to counteract muscle loss [97]. These recommendations are based on observational data or studies in non-critically ill patients [99,100]. Therefore, this review acknowledges the need of further research to consolidate the most efficacious in-unit and post-discharge nutrition and physical activity strategies with well conducted clinical trials [97].

Vitamin D deficiency is common in the elderly and the critically ill and is associated with increased mortality. According to the 2019 ESPEN guidelines, vitamin D can be supplemented (GPP) with a single dose of 500,000 IU vitamin D3 within one week after admission (grade 0) in case of measured low plasma levels (25-hydroxy vitamin D <12.5 ng/mL, or <50 nmol/L) [17].

#### 4.1.3. Other Risk Groups

With the development of advanced medicine, the new patient group of so-called chronic critically ill patients has emerged and is often treated in the step-down unit. A recent review provides a thorough discussion of the nutritional approach for these patients [101]. The ASPEN guidelines, based on expert consensus, define chronic critically ill patients as having persistent organ dysfunction requiring ICU treatment >21 days and suggest management with aggressive high-protein EN therapy and, when feasible, use of a resistance exercise program [27].

Patients with renal failure can be placed on a standard enteral formula following the recent ICU recommendations for protein administration [17,27]. According to the ASPEN guidelines, a special formula might be indicated in case of significant electrolyte or volume abnormalities. Renal replacement therapy may require increased protein support (≤2.5 g/kg/day) (grade very low) and adapted micronutrient support [17,27]. Importantly, protein support should not be restricted in order to avoid or delay initiation of dialysis therapy [27].

The ESPEN Society provides separate nutritional management recommendations for severe burn patients [102] and for patients with acute or chronic liver failure [103].

Patients with alcohol abuse are particularly at risk for thiamine deficiency, as are patients with severe sepsis, congestive heart failure, or burn injuries [104]. Recommended daily doses for alcoholic patients are substantially higher than for non-alcoholic patients, reaching up to 500 mg thiamine three times daily [105]. Another review underlines the importance of additive therapies such as correction of electrolyte disturbances and malnourishment [106].

### 4.2. International Guidelines on Nutrition in Critically Ill Patients

In this review on most recent practices to provide adequate nutrition in the critically ill, we concentrated on three widely used and regularly updated international guidelines issued by the Canadian Critical Care Society/Canadian Critical Care Trials Group (CCCS/CCCTG) [48] in 2015, by ASPEN [27] in 2016, and, most recently, by ESPEN [17]. These three guidelines discuss main and subtopics of critical care nutrition in which most recommendations are based on expert consensus (Table S1). As such, ESPEN guidelines include the most recent studies and meta-analyses from robust RCTs. Moreover, recommendations have been well separated into grade A, B, level 0, or GPP, according to the strength of the evidence [107]. The ASPEN guidelines include the largest number of recommendations, whereas certain aspects of the guidelines are not exclusively covered by the European and Canadian societies (ESPEN; CCCS/CCTG) (e.g., non-intubated patients (ESPEN), ornithine ketoglutarate in burn patients or specific mentioning of vitamin C supplementation (CCCS/CCTG), or recommendation on insulin administration (ESPEN; CCCS/CCTG). Many recommendations in the ASPEN guidelines target a certain group of critically ill, whereas the ESPEN and CCCS/CCCTG guidelines address the critically ill in general (e.g., vitamin D supplementation). Notably, ASPEN proposes a higher number of recommendations for subgroups of critically ill patients by labelling with a grade of expert consensus due to the lack of solid evidence. Moreover, the concept of ALI (acute lung injury), not part of the 2013 Berlin definition of acute respiratory distress syndrome (ARDS), is not mentioned in the ESPEN guidelines [28]. ASPEN and ESPEN offer recommendations for frequent diseases seen in critical care, such as sepsis and acute pancreatitis and statements for trauma and burn patients. In addition, the American society includes a chapter on end-of-life care.

### 4.3. Medical Nutrition Therapy: EN, PN, ONS, and Combinations

Routes, quantity, and timing of nutritional delivery have been widely discussed over recent decades. With the increasing administration of parenteral nutrition (PN) to the critically ill, some studies have shown more infectious complications in comparison to enteral nutrition (EN) [29,30]. Enteral feeding was subsequently used in first line with an important reduction in the use of PN [108]. However, achieving energy and protein targets with EN alone is difficult during the ICU and IMC-U stay and may result in undernutrition in most patients [31,32]. Recent RCTs have shown no increase in the rate of complications independent of the chosen route of nutrition [57,109]. Avoiding under- or overnutrition is more important than the feeding route [108].

The SPN study (*n* = 305) showed that the use of supplemental parenteral nutrition (SPN) (when EN was insufficient to match the energy targets at day 4 after ICU admission) could reduce nosocomial infections after the end of the intervention and until the end of the observation period (days 9 to 28), as well as antibiotic use during the ICU stay [7]. However, no impact on infections was shown when counting the infection rate from the randomization day onward. The TICACOS pilot study (*n* = 130) [55] showed, based on prospective intention to treat analysis of the data, that IC-guided nutrition therapy provoked an increase in complications with more infections, longer duration of ventilation, and prolonged ICU stays. A per protocol analysis, on the contrary, suggested a short-lived survival benefit [55].

Some other studies (e.g., EAT-ICU (*n* = 203) [56], TARGET (*n* = 3957) [11], EDEN (*n* = 1000) [13]) showed no difference between the early versus standard initiation groups in primary clinical outcomes, but secondary outcomes revealed hyperglycemia [13,56] and decreased gastric tolerance [11,13] in the early nutrition intervention group. The PermiT trial (*n* = 894) group studied permissive underfeeding (40–60% of calculated caloric requirements) compared to standard enteral feeding (70–100%) with similar protein intake. The results showed no difference in 90-day mortality and no differences in other significant outcomes between the groups [12]. The results of the EPaNIC trial (*n* = 4640) revealed no difference in 90-day mortality but an increased complication rate with early initiation of parenteral support (i.e., infections, longer mechanical ventilation, and length of stay) [54].

The divergence of results may be related to the heterogeneous patient population and highly variable study designs with differences such as the amount of calories administered, the timing of EN or PN initiation, and the use of IC or predictive formulas.

Considering these contradictory findings, expert guidelines agree on the following indications for MNT [17,27]: (1) Oral nutrition should be preferred over EN or PN in patients able to eat voluntarily (GPP). (2) If needed, oral nutritional supplements (ONS) can be given with the aim to achieve energy and protein needs [34]. (3) Early EN (within 48 h) is suggested for all patients with a functional digestive tract who cannot eat voluntarily (grade B). Enteral feeding can be performed progressively after the early phase of the critical illness and, after 3 days, caloric delivery can be increased up to 80–100% of the measured EE (grade 0). (4) In patients who do not tolerate full EN during the first week, initiation of SPN should be considered on a case-by-case basis and should only be started after all strategies to maximize EN tolerance have been attempted (GPP). (5) In case of impossible oral feeding and EN contraindications, PN must also be started progressively during the first week of the ICU stay.

The timing of PN initiation is a highly debated topic. ESPEN experts propose an early implementation of PN within 3–7 days in case of contraindications to oral feeding or EN (grade B). SPN should be considered for patients who do not tolerate full-dose EN during the first week of ICU or IMC-U stay (GPP). In contrast, the ASPEN guidelines recommend SPN in case of insufficient early EN (<60% of energy and protein requirements) only after 7–10 days. Further, the ESICM guidelines extensively discuss clinical states and medications that warrant delayed EN initiation [17,27,110].

### 4.4. Adaptation/Reassessment and Monitoring

Monitoring is an essential part of nutritional management and is central to achieving high-quality nutritional care. The quantity of prescribed and received nutrition in terms of volume, energy, and protein must be assessed including non-nutritional calories (e.g., Propofol^®^, citrate) [35] in order to avoid under- or overfeeding. Gastric tolerance must be monitored, including clinical abdominal examination and evaluation of stools, gastric residual volume, or vomit [111]. Laboratory monitoring of glycemia, electrolytes, liver parameters, and triglycerides is necessary to assess MNT tolerance [35]; prealbumin can be helpful to assess the efficacy of the MNT [36].

### 4.5. Role of the Intensive Care Unit Dietician

Even if nutritional guidelines and local protocols have been shown to be helpful in standardizing and improving the quality of MNT in ICUs [37,38], physicians and nurses have reported a lack of knowledge, training, and time in nutrition management [39,40]. Dieticians, specialized in ICU nutrition, can provide valuable help concerning nutrition management for the ICU teams. It was suggested that the presence of dieticians in the ICU team was helpful to achieve the desired daily energy and protein targets during the ICU stay [39]. Their role should also include detection of too early aggressive nutrition support with the aim to prevent refeeding and overfeeding. To increase the quality of the care, we recommend working in a multidisciplinary team including intensivists, ICU nutritional specialists, dieticians, nurses, physiotherapists, and others if necessary. In addition, regular adaptation of local protocols to the current guidelines and facilitating protocol adherence is necessary [41]. These efforts should be extended to patients on the IMC-U.

### 4.6. Continuation of Medical Nutrition Therapy After Transfer or Discharge to Home

MNT is not only important during ICU/IMC-U stay, it is an essential part of both the post-ICU/IMC-U recovery phase and after hospital discharge [43]. While advances in medicine and improvements in the quality of care have reduced ICU mortality in recent years, the number of post-critically ill patients in rehabilitation has consequently increased [112]. Low oral intake (e.g., only 700 kcal/day for one week after extubation) has been reported to be an amount insufficient to cover the energy needs of 97% of patients [42,76]. In this context, most critically ill patients would need continuous medium-length or long-term MNT (ONS, EN, PN, and SPN) coupled with exercise for rehabilitation. MNT can also be continued at home after hospital discharge. However, it is also important to consider the physical and mental abilities of patients and their relatives, in addition to the patient’s living conditions, with the aim of achieving a supportive environment [44]. Educating the patient and his/her close relatives should be proposed to achieve the highest level of independence possible [44]. Follow up involving a multidisciplinary nutrition team helps to increase the quality of care, facilitating the adaptation of the therapy and the support in case of possible complications [44].

## 5. Complications

### 5.1. Over- and Underfeeding

Over- and underfeeding frequently occur in critical care patients as nutritional substrates, whether given parenterally or enterally, do not have the same metabolic consequences in the critically ill. While overfeeding occurs more often with PN, underfeeding is more common in enterally fed patients. Patients most at risk for energy excess or nutritional deficits are those with low caloric targets (e.g., due to small body size, non-nutritional energy (e.g., Propofol^®^ for sedation) or geriatric patients) [113].

Overfeeding is defined as the administration of excess energy in relation to the body’s need to maintain metabolic homeostasis when exceeding >110% of the EE [17] and can lead to a multitude of complications and increased mortality. Depending on the macro-nutrients delivered in excess, these complications include hypercapnia, refeeding syndrome (RFS) and liver disturbances, hyperglycemia and hypertriglyceridemia, as well as azotemia, hypertonic dehydration, and metabolic acidosis [114]. Therefore, higher protein amounts (1.3 g/kg/day) can be delivered progressively as long as these amounts are not associated with overfeeding (grade 0) [17].

The lower limit of optimal energy supplementation to the critically ill is not yet clearly defined and may vary depending on the underlying disease and nutritional status of the patient. Underfeeding (<70% of the defined target) [17] often occurs during EN due to feeding intolerance, airway management, or disruption of feeding due to elective procedures [115].

### 5.2. Refeeding Syndrome

RFS is recognized as a life-threatening cause-independent complication in severely malnourished patients. These patients may show severe adverse reactions involving the cardiac, respiratory, hematological, hepatic, and neuromuscular systems when refed too rapidly, especially with carbohydrates [46]. The difficulty to precisely define RFS limits screening and estimated numbers of unreported cases are presumably high. Therefore, assessing the risk of RFS before starting MNT in high-risk patients [46,47], including those with anorexia nervosa, chronic alcoholics, elderly patients with multiple comorbidities, patients with signs of chronic malnutrition (e.g., marasmus, severe inflammatory bowel disease, short bowel syndrome), the morbidly obese, and patients receiving PN, has been suggested [47]. Furthermore, an algorithm for RFS management has been proposed, including an initial risk assessment and recommendations for electrolyte and fluid repletion [116,117]. However, a more recent retrospective study [118] in critically ill patients with prolonged ventilation found no baseline characteristics predicting RFS. Patients developing RFS had a reduced 6-month mortality risk when receiving low caloric intake. These results confirm those of a previous trial proposing a caloric restriction in the context of RFS [119] as follows: (1) Reduced energy intake of 20 kcal/h for at least 2 days, (2) if serum phosphate concentrations did not need to be supplemented, as determined per protocol (as described in the Appendix S3 of [119]), energy intake should be returned to normal during 2–3 days, (3) a gradual return to 40 kcal/h for 24 h, then increased goals to 60 kcal/h for 24 h, followed by 80% of calculated energy goals for another 24 h, with 100% of goals achieved by day 4 [119].

According to the National Institute for Health and Care Excellence (NICE) guidelines, patients at risk should be nourished with regular control of electrolytes as follows: (1) Feeding starting at 10 kcal/kg/day, (2) increasing to full need over ≥4 days, and (3) supplementation of thiamine (vitamin B1, 200–300 mg/day) for the first 10 days [47,120].

### 5.3. Parenteral Nutrition

Complications attributed to the use of PN include mechanical complications with catheter obstruction, catheter infections, venous thrombosis, or pneumothorax [121]. Short-term metabolic complications can occur as well as fluid imbalance (overload or dehydration), electrolyte disturbances, glycemic abnormalities, and dyslipidemia [122]. These complications can be avoided by systematic and precise monitoring [122]. Major long-term metabolic complications include metabolic bone diseases and hepatobiliary disorders. It was shown that the frequency of osteoporosis and osteomalacia increases in patients with long-term PN [123]. In addition, intestinal failure-associated liver disease can occur during parenteral feeding, and cholestasis and hepatic steatosis in those with long-term PN. Hepatobiliary disorders have been attributed to overfeeding during PN [121,124].

Some recent studies suggest that, if provided in isocaloric doses, PN and EN result in similar clinical outcomes [7,57,109]. Therefore, PN is not in opposition to EN but can be used as an additional therapy as soon as a close monitoring of the MNT is assured [39,108]. However, PN should not be started before all strategies to maximize EN tolerance have been attempted (GPP) [17].

### 5.4. Enteral Nutrition

Two large randomized trials specifically designed to investigate the feeding route in critically ill patients [57,109] found more episodes of both hypoglycemia and vomiting with EN compared to PN. Furthermore, the NUTRIREA-2 trial assessing severely ill ICU patients under mechanical ventilation and on vasopressors detected a pronounced difficulty to reach protein and calorie targets and an increased risk for gastrointestinal complications (e.g., diarrhea, bowel ischemia, acute colonic pseudo-obstruction) with early EN [57].

#### Enteral Nutrition and Dysphagia: Risk of Aspiration, Swallowing Screening

Dysphagia occurs in 50–70% of the critically ill [125]. Risk factors include older age, congestive heart failure, sepsis, perioperative stroke, non-coronary bypass surgical procedures, transesophageal echocardiography, and previous stroke [125]. Patients suffering from dysphagia are at major risk for aspiration. Several high-quality swallowing tests (e.g., DePippo, Horner, Kidd) developed to thoroughly assess aspiration risk have been summarized in a recent review [126] in which the authors report the swallowing screening published by Daniels et al. in 1998 to be the most convenient. This assessment is also applicable for intubated patients for a certain period of time. Moreover, it is considered by non-speech therapists (i.e., nurses specialized in intensive care) to be the most suitable assessment.

In case of aspiration risk, feeding tubes are used to nourish the patient. Tube selection depends on the expected duration of feeding. Furthermore, the selection of a large bore tube (≥14 French) ensures gastric suctioning and decompression in case of gastroparesis and paralytic ileus, which are usually present during the early postoperative period.

The measurement of gastric residual volume (GRV) for the assessment of gastrointestinal function is commonly used and may help to detect intolerance to EN during initiation and progression of EN, especially in patients who underwent abdominal procedures or major surgery. However, monitoring of GRV in case of established EN may not be necessary. The ESPEN guidelines suggest that EN should be delayed when GRV is >500 mL/6 h, and prokinetic treatment should be considered if acute abdominal complications are excluded [17]. ASPEN and the Surviving Sepsis Initiative support the use of prokinetics (e.g., metoclopramide (three times 10 mg/day) and erythromycin (3–7 mg/kg/day)) in the case of feeding intolerance [27,127].

## 6. Financial Aspects

Disease-related malnutrition has relevant consequences on patient outcome, especially in the most severely ill patients treated on ICUs and IMC-Us. However, for several reasons it remains difficult to demonstrate the economic benefit related to improved outcome rooted in MNT. The comparison of different clinical practices in hospitals and countries, variable costs for hospital staff and MNT products, and heterogeneous accounting systems complicate any reliable comparison of cost and benefit. Furthermore, the acquisition of nationwide and international data is limited and explains why study data are scarce [128] and why cost-economic analyses show discrepancies in this important field of interest.

Relevant data for the critically ill exist concerning the potentially cost-saving introduction of the pre-mixed multi-chamber bag compared to compound PN. In a real-world analysis of PN use, potential savings of up to $1545 USD per patient using a multi-chamber bag could be achieved according to US data from the Premier Perspective™ database [129]. Patients on PN also benefit from a nutritional care team in order to assure adequate indication, application, and adaptation of PN, resulting in savings of 245,000 € EUR in one year per 100 treated patients in a Swiss university hospital [130]. A meta-analysis involving six clinical trials evaluating early EN in the ICU setting found a statistically significant reduction in mortality compared to standard timing of EN [131]. A cost-effectiveness analysis of these data showed a $14,462 USD reduction in total costs per patient for acute hospital care of patients receiving EN within 24 h after admission. These results were robust also when including sensitivity analysis with European cost data [132].

The Swiss SPN study added PN on days 4–8, whenever >60% of the caloric needs where not covered by EN on day 3. Based on the Swiss accounting system (Swiss DRG), a simulated calculation revealed a risk reduction for infection of 10%, resulting in savings of 63,048 CHF per infection [7,133]. The cost-analysis of the EPaNIC trial showed increased expenses with early-PN, mainly due to pharmacy-related costs and higher expenditures for PN and anti-infectious agents when compared to late PN patients who only received PN when EN remained insufficient after the first week of ICU treatment. Withholding early PN resulted in a net cost saving of 1210 Euro per patient [134].

The pronounced loss of lean body tissue, lack of mobilization, and increased protein energy needs all lead to higher complication rates with increased morbidity and mortality in this most fragile group of critically ill patients. Further studies, such as the recently published EFFORT study [135], should describe the effect of MNT not only in terms of clinical benefit but also with regard to monetary equivalent. Such data are of utmost interest to support and spread the relevance of MNT and linked-care teams.

## 7. Conclusions

MNT in all categories of critically ill patients remains a challenge, not only because these patients suffer from a pronounced stress response and are at higher risk for complications, but also due to their heterogeneity and their different illness phases (acute, subacute, chronic critically ill; pre- or post- resuscitation). Further studies are urgently needed to answer important questions such as the best timing, route, dose, and composition of MNT for the critically ill and to determine how to assess and to adapt to each patient’s individual needs.

Current guidelines, as presented and discussed in this review, integrate actual knowledge and best clinical practice. Further steps would be to introduce them into (pre-existing) local feeding protocols. A MNT team offers an important additional value to optimize both patient care and education of hospital staff.

## Supplementary Materials

The following are available online at https://www.mdpi.com/2077-0383/8/9/1395/s1, Table S1: Overview of current guidelines issued by ESPEN, ASPEN and CCCS/CCCTG.
