# Supplementary material for: Medical Nutrition Therapy in Critically Ill Patients Treated on Intensive and Intermediate Care Units: A Literature Review
Source: J Clin Med. 2019 Sep 6;8(9):1395. doi: 10.3390/jcm8091395 (PMC6780491; doi:10.3390/jcm8091395)
Supplement: Supplementary file 1 [file jcm-08-01395-s001.pdf]

Table 1. Comparison of ASPEN, ESPEN and CCCS/CCCTG guidelines for nutrition in critically ill patients.

#			ASPEN 2016 [27]			ESPEN 2018 [17]		CCCS/ CCCTG 2015 [27]
	# recommendations		76*)			57**)		46**)
		LOE			LOE			
				**A) NUTRITION ASSESSMENT***)**				
1	Determination of nutrition risk (nutritional risk screening [NRS 2002], NUTRIC score) in all patients admitted to the ICU for whom volitional intake is anticipated to be insufficient. High nutrition risk identifies those patients most likely to benefit from early EN therapy.	EC	A1	A general clinical assessment should be performed to assess malnutrition in the ICU until a specific tool has been validated	GPP – SC	R2		
2	Nutrition assessment is suggested to include an evaluation of comorbid conditions, function of GI tract and risk of aspiration	EC	A2					
3	It is suggested to determine energy requirements by 1) IC 2) Published predictive equation or simplistic weight-based equation (25-30 kcal/kg/d) in absence of IC	Very low	A3a, A3b	In critically ill mechanically ventilated patients, EE should be determined using IC; if calorimetry is unavailable, VO_2 from pulmonary arterial catheter or VCO_2 from the ventilator will give a better evaluation on EE than predictive equations	B; cons.	R15	Data are insufficient to make a recommendation on the use of indirect calorimetry vs. predictive equations to determine energy needs for nutrition or to guide when nutrition is to be supplemented in the critically ill	R3.1

4	Ongoing evaluation of adequacy of protein provision is suggested	EC	A4					
							In patients receiving PN or EN, data are insufficient to make a recommendation regarding the use of intravenous supplementation with higher amounts of branched-chain amino acids in the critically ill	R9.1
				Blood glucose should be measured initially (after ICU admission or after artificial nutrition initiation) and at least every 4h, for the first two days in general	GPP – SC	R53		
				Insulin shall be administered, when glucose levels >10 mmol/l	A	R54		
				Electrolytes (potassium, magnesium, phosphate) should be measured at least once daily for the first week	GPP – SC	R55		
				NON-INTUBATED PATIENTS				
				In those not reaching the energy target on an oral diet, oral nutritional supplements should be considered first and then EN	GPP – SC	R41		
				In those with dysphagia, texture-adapted food can be considered; if swallowing is proven unsafe, EN should be administered	GPP – SC	R42		

				In those with dysphagia and a very high aspiration risk, postpyloric EN or, if not possible, temporary PN during swallowing training with removed nasoenteral tube can be performed	GPP – SC	R43		
**B) INITIATION OF EN**								
5	Initiation of early EN within 24-48 hours is recommended	Very low	B1	Medical nutrition shall be considered for all patients staying in the ICU, mainly for more than 48 hours<br><br>Oral diet shall be preferred over EN or PN in critically ill patients who are able to eat<br><br>Early EN should be performed in patients<br>- receiving ECMO<br>- with TBI<br>- with stroke (ischaemic or haemorrhagic)<br>- with spinal cord injury<br>- with severe acute pancreatitis<br>- after GI surgery<br>- after abdominal aortic surgery<br>- with abdominal trauma when the continuity of the GI tract is confirmed/restored<br>- receiving neuromuscular blocking agents	GPP – SC<br><br>GPP – SC<br><br>B	R1<br><br>R3<br><br>R40	Based on 16 level-2 studies, we recommend early EN (within 24-48 hours following ICU admission in the critically ill	R2

				- managed in prone position<br>- with open abdomen<br>- with diarrhea, regardless of the presence of bowel sounds unless bowel ischaemia or obstruction is suspected				
6	EN over PN is suggested	Low to very low	B2	If oral intake is not possible, initiate early rather than late EN (≤48 h) in critically ill adults<br><br>If oral intake is not possible, initiate early EN (within 48 hours) rather than early PN shall in critically ill adults	B<br><br><br><br>A	R4<br><br><br><br>R5	Based on 16 level-2 and 1 level-1 study, when considering nutritional support for the critically ill, we recommend the use of EN over PN in patients with an intact gastrointestinal tract	R1
7	GI contractility factors should be evaluated when initiating EN; overt signs of contractility should not be required prior to initiation of EN	EC	B3				Based on 1 level-1 study, 3 level-2 studies and 2 cluster RCTs, when starting EN in critically ill patients, strategies to optimize delivery of nutrients (starting at target rate, volume-based feeding strategies, higher threshold of gastric residual volumes, use of prokinetics, concentrated feeding solutions and small bowel feedings) should be considered	R3.2
8	In case of high risk for aspiration or intolerance to gastric EN, level of infusion is recommended to be diverted lower in the GI tract; in most critically ill patients it is acceptable to initiate EN in the stomach	Moderate to high	B4a, B4b					
9	In the setting of haemodynamic compromise or instability, EN should be withheld until the patient is fully	EC	B5	EN should be delayed<br>- if shock is uncontrolled and haemodynamic and tissue perfusion goals are unmet whereas low-dose	B	R38		

	resuscitated and/or stable. Initiation/reinitiation of EN may be considered with caution in patients undergoing withdrawal of vasopressor support.			EN can be started as soon as shock is controlled with fluids and vasopressors/inotropes, while remaining vigilant for signs of bowel ischaemia;<br>- in case of uncontrolled life-threatening hypoxaemia, hypercapnia or acidosis, whereas EN can be started in patients with stable hypoxaemia, and compensated or permissive hypercapnia and acidosis;<br>- in patients suffering from active upper GI bleeding, whereas EN can be started when the bleeding has stopped and no signs of re-bleeding are observed;<br>- in patients with overt bowel ischaemia;<br>- in patients with high-output intestinal fistula if reliable feeding access distal to the fistula is not achievable;<br>- in patients with abdominal compartment syndrome; and<br>- if gastric aspirate volume is above 500 ml/6h				

**C) DOSING OF EN**								
10	It is suggested that patients who are at low nutritional risk with normal baseline nutritional status and low disease severity (e.g. NRS 2002 ≤3 or NUTRIC score ≤5) who cannot maintain volitional intake do not require specialized nutrition therapy over the first week in the ICU	EC	C1				Based on 4 level-2 studies, intentional underfeeding of calories (not protein) should be considered in patients at low nutritional risk. However, this recommendation does not apply to patients at high nutritional risk	R3.3b
11	Either trophic or full nutrition by EN is appropriate for patients with ARDS/ALI and those expected to have a duration of mechanical ventilation ≥72 hours, as these 2 strategies of feeding have similar patient outcomes over the first week of hospitalization.	High	C2	Low dose EN should be administered in patients<br>- receiving therapeutic hypothermia and increasing the dose after rewarming<br>- with intra-abdominal hypertension without abdominal compartment syndrome, whereas temporary reduction or discontinuation of EN should be considered when intra-abdominal pressure values further increase under EN<br>- with acute liver failure when acute, immediately life-threatening metabolic derangements are controlled with or without liver support strategies,	B	R39	Based on 2 level-1 studies, in patients with ALI, an initial strategy of trophic feeds for 5 days should not be considered	R3.3a

				independent of grade of encephalopathy				
12	Patients at high nutritional risk (e.g. NRS 2002 ≥5 or NUTRIC score ≥5 without interleukin 6) or severely malnourished should be advanced toward goal as quickly as tolerated over 24-48 hours while monitoring for refeeding syndrome; efforts to provide >80% of estimated or calculated goal energy and protein within 48-72 h should be made to achieve the clinical benefit of EN over the first week of hospitalization	EC	C3	To avoid overfeeding, early full EN or PN shall not be used in critically ill patients but shall be prescribed within 3-7 days<br><br>If IC is used, isocaloric rather than hypocaloric nutrition can be progressively implemented after the early phase of acute illness<br><br>Hypocaloric nutrition (<70% of EE) should be administered in the early phase of acute illness<br><br>After day 3, caloric delivery can be increased up to 80-100% of measured EE<br><br>If predictive equations are used to estimate energy need, hypocaloric nutrition (<70% estimated needs) should be preferred over isocaloric nutrition for the first week of ICU stay	A<br><br>0<br><br>B<br><br>0<br><br>B	R8<br><br>R16<br><br>R17<br><br>R18<br><br>R19		
13	Sufficient (high-dose) protein should be provided; protein requirements are expected to be in the range of 1.2-2.0 g/kg ABW per day and may likely be even higher	Very low	C4	During critical illness, 1.3 g/kg/day protein equivalents can be delivered progressively	0	R22	Data are insufficient to make a recommendation regarding the use of high protein diets or escalating doses of protein in critically ill patients	R4.2c<br><br>R4.3

	in burn or multitrauma patients						Based on 5 level-2 studies, when initiating enteral feeds, the use of whole protein formulas (polymeric; not peptides) should be considered	
				The amount of glucose (PN) or carbohydrates (EN) administered to ICU patients should not exceed 5 mg/kg/min	GPP – SC	R23		
**D) MONITORING TOLERANCE AND ADEQUACY OF EN**								
14	Patients are suggested to be monitored daily for tolerance of EN; inappropriate cessation of EN is suggested to be avoided; ordering a feeding status of NPO for the patient surrounding the time of diagnostic tests or procedures should be minimized to limit propagation of ileus and to prevent inadequate nutrient delivery	EC	D1					
15	GRVs are suggested not to be used as part of routine care to monitor ICU patients receiving EN; for those ICUs where GRVs are still utilized, holding EN for GRVs <500 ml in the absence of other	Low	D2a, D2b				Based on 3 level-2 studies, a gastric residual volume of 250-500 ml and frequency of checking residuals either q4 or q8 hours should be considered to optimize delivery of EN in the critically ill.	R5.5

	signs of intolerance should be avoided							
							Data are insufficient to make a recommendation to return gastric residual volumes to a certain threshold in critically ill adults. Based on 1 level-2 study, re-feeding GRV ≤250 ml or discarding GRV may be acceptable	R5.6
16	Enteral feeding protocols are recommended to be designed and implemented to increase the overall percentage of goal calories provided; use of a volume-based feeding protocol or a top-down multistrategy protocol are suggested to be considered	Moderate to high	D3a, D3b				Based on 2 level-2 studies and 3 cluster RCT, a feeding protocol should be considered that incorporates strategies to optimize delivery of EN in critically ill adults	R5.1
17	Patients receiving EN should be assessed for risk of aspiration; steps to reduce this risk of aspiration and aspiration pneumonia should be proactively employed<br>- Level of feeding is recommended to be diverted by postpyloric enteral access device placement in patients deemed to be at high risk for aspiration	EC	D4, D4a, D4b, D4c, D4d	Continuous rather than bolus EN should be used<br><br>Gastric access should be used as the standard approach to initiate EN<br><br>Postpyloric feeding should be used in patients with gastric feeding intolerance unresolved using prokinetics<br><br>In patients deemed to be at high risk for aspiration,	B<br><br>GPP – SC<br><br>B<br><br>GPP – SC	R9<br><br>R10<br><br>R11<br><br>R12	Data are insufficient to make a recommendation regarding the use of low pH feeds in the critically ill.<br><br>Based on 1 level-1 study and 5 level-2 studies, in critically ill patients who experience feed intolerance (high gastric residuals, emesis), we recommend the use of a promotility agent. Given safety concerns associated with erythromycin, this	R4.4<br><br>R5.2

	- For high-risk patients or those shown to be intolerant to bolus gastric EN, delivery of EN should be switched to continuous infusion<br>- In patients at high risk of aspiration, agents to promote motility, such as prokinetic medication (metoclopramide or erythromycin), are suggested to be initiated where clinically feasible<br>- Nursing directives to reduce risk of aspiration and VAP are suggested to be employed; in all intubated ICU patients receiving EN, the head of the bed should be elevated 30-45° and use of chlorhexidine mouthwash twice a day should be considered			postpyloric, mainly jejunal feeding can be performed<br><br>In critically ill patients with gastric feeding intolerance, intravenous erythromycin should be used as a first line prokinetic therapy<br><br>Alternatively, intravenous metoclopramide or a combination of metoclopramide and erythromycin can be used as a prokinetic therapy	B<br><br>0	R13<br><br>R14	recommendation is made for metoclopramide. Data are insufficient recommend combined use of metoclopramide and erythromycin.<br><br>Based on 16 level-2 studies, small bowel feeding compared to gastric feeding may be associated with a reduction in pneumonia in critically ill patients. In units where small bowel access is feasible, we recommend the routine use of small bowel feedings. In units where obtaining access involves more logistical difficulties, small bowel feedings should be considered in those at high risk for intolerance to EN (on inotropes, continuous infusion of sedatives, or paralytic agents, or in patients with high nasogastric drainage) or at high risk for regurgitation and aspiration (nursed in supine position). Finally, when small bowel access is not feasible (no access to fluoroscopy or endoscopy and blind techniques not reliable), small bowel feedings should be considered for select patients	5.3

							who repeatedly demonstrate high gastric residuals and do not tolerating adequate amounts of EN intragastrically<br><br>Based on 1 level-1 and 1 level-2 study, we recommend elevating the head of the bed 45° critically ill patients receiving EN. If not possible, attempt to raise the head of the bed as much as possible should be considered<br><br>Data are insufficient to recommend administering EN via closed vs. open system in the critically ill<br><br>Data are insufficient to recommend EN administered continuously vs. other methods of administration in the critically ill<br><br>There are insufficient data to make a recommendation on gastrostomy feeding vs. nasogastric feeding in the critically ill	 <br><br><br><br>R5.4<br><br><br><br><br><br><br><br><br><br>R6.1<br><br><br><br><br>R6.3<br><br><br><br><br><br>R6.4
18	Neither blue food coloring nor any coloring agent is suggested to be used as marker for aspiration of EN; glucose oxidase strips	EC	D5					

	are not suggested to be used as surrogate markers for aspiration in the critical care setting							
19	EN is not suggested to be automatically interrupted for diarrhea, rather feeds are suggested to be continued while evaluating the etiology of diarrhea in ICU patients to determine appropriate treatment	EC	D6					
**E) SELECTION OF APPROPRIATE ENTERAL FORMULATION**								
20	A standard pyloric formula is suggested to be used when initiating EN in the ICU setting; avoiding routine use of all specialty formulas in critically ill patients in a MICU and disease-specific formulas in the SICU is suggested	EC	E1				Data are insufficient to recommend high fat/low CHO diets for critically ill patients<br><br>Data are insufficient to recommend low fat/high CHO diets for critically ill patients<br><br>Data are insufficient to recommend low carbohydrate diets in conjunction with insulin therapy for critically ill patients	R4.2a<br><br>R4.2b<br><br>R10.4b
21	Immune-modulating enteral formulations (arginine with other agents, including EPA, DHA, glutamine, and nucleic acid) should not be used routinely in the MICU; considerations for these formulations should be reserved for patients	Very low	E2				Based on 5 level-1 studies and 22 level-2 studies, we do not recommend diets supplemented with arginine and other select nutrients for critically ill patients	R4.1a

	with TBI and perioperative patients in the SICU							
22	Routine use of an enteral formulation characterized by an anti-inflammatory lipid profile (e.g. omega-3 FOs, borage oil) and antioxidants in patients with ARDS and severe ALI. Given conflicting data, a recommendation cannot be made	Low to very low	E3	High doses of omega-3-enriched EN formula should not be administered as bolus<br><br>EN enriched with omega-3 fatty acids within nutritional doses can be administered<br><br>High doses omega-3 enriched enteral formulas should not be given on a routine basis<br><br>Antioxidants should not be administered as high-dose monotherapy without proven deficiency	B<br><br>0<br><br>B<br><br>B	R30<br><br>R31<br><br>R32<br><br>R35	Based on 3 level-1 studies and 5 level-2 studies, use of an enteral formula with FOs, borage oils and antioxidants in patients with ALI and ARDS should be considered<br><br>Data are insufficient to recommend supplementation of fish oils alone in critically ill patients	R4.1b(i)<br><br>R4.1b(ii)
23	Commercial mixed fiber formula is suggested not to be used routinely in adult critically ill patients prophylactically to promote bowel regularity or prevent diarrhea. Use of a commercial mixed-fibre-containing formulation is suggested to be considered if there is evidence of persistent diarrhea; both soluble and insoluble fiber is suggested to be avoided in patients at high risk for bowel ischaemia or severe	Low	E4a, E4b				Data are insufficient data to support the routine use of fibre (soluble or insoluble) in enteral feeding formulas in critically ill patients	R4.5

	dysmotility. Use of small peptide formulations is suggested to be considered in patients with persistent diarrhea, suspected malabsorption or lack of response to fiber							
**F) ADJUNCTIVE THERAPY**								
24	A fermentable soluble fiber additive is suggested to be considered for routine use in all hemodynamically stable MICU/SICU patients placed on a standard enteral formulation; 10-20 g of a fermentable soluble fiber supplement is suggested to be given in divided doses over 24 h as adjunctive therapy if there is evidence of diarrhea	EC	F1					
25	While the use of studied probiotics species and strains appear to be safe in general ICU patients, they should be used only for select medical and surgical patient populations for which RCTs have documented safety and outcome benefit – no recommendation is possible at this time	Low	F2				Based on 4 level-1 studies and 24 level-2 studies, the use of probiotics should be considered in the critically ill	R6.2

26	A combination of antioxidant vitamins and trace minerals in doses reported to be safe in critically ill patients is suggested to be provided to those who require specialized nutrition therapy	Low	F3				Based on 8 level-1 and 19 level-2 studies, we do not recommend the use of supplemental combined vitamins and trace elements in critically ill patients<br><br>Data are insufficient to make recommend vitamin C supplementation in critically ill patients	R11.1<br><br>R11.3
27	Supplemental enteral glutamine is suggested not to be routinely added to an EN regimen in critically ill patients	Moderate	F4	Additional enteral GLN should not be administered to ICU patients except for those hospitalized for burns and trauma	B	R28	Based on 3 level-1 and 8 level-2 studies, we recommend that enteral glutamine NOT be used in critically ill patients<br><br>Based on 1 level-1 and 1 level-2 study, we recommend that high dose combined parenteral and enteral glutamine supplementation NOT be used in critically ill patients<br><br>Data are insufficient to recommend the use of enteral glutamine vs. parenteral dipeptide supplementation. However given concerns of glutamine supplementation in general as per sections 4.1c EN glutamine, 9.4a PN glutamine and 9.4b EN+PN glutamine, we strongly recommend that glutamine supplementation NOT be used	R4.1c<br><br>R9.4b<br><br>9.4c

							in critically ill patients, hence we do not recommend the use of enteral glutamine or parenteral dipeptides.	
							Data are insufficient to recommend ß Hydroxyl Methyl Butyrate (HMB) supplementation in critically ill patients.	R6.5
**G) WHEN TO USE PN**								
28	In patients at low nutrition risk (e.g. NRS 2002 ≤3 or NUTRIC score ≤5), exclusive PN is suggested to be withheld over the first 7 days following ICU admission if the patient cannot maintain volitional intake and if early EN is not feasible	Very low	G1	In case of contraindications to oral or EN, PN should be implemented within 3-7 days<br><br>PN should not be started until all strategies to maximise EN tolerance have been attempted	B<br><br><br><br>GPP - SC	R6<br><br><br><br>R21	Based on 1 level-1 and 7 level-2 studies, we recommend that PN not be started at the same time as EN in critically ill patients starting on EN. In patient's not tolerating adequate EN, data are insufficient to recommend when PN should be initiated. Practitioners need to weigh the safety and benefits of initiating PN in patients not tolerating EN on a case-by-case basis. We recommend that PN not be started in critically ill patients until all strategies to maximize EN delivery (such as small bowel feeding tubes, motility agents) have been attempted.	R7.1
29	In patients at high nutritional risk (e.g. NRS 2002 ≥5 or NUTRIC score ≥5) or severely malnourished, exclusive PN is suggested to be	EC	G2	In severely malnourished patients, early and progressive PN can be provided instead of no nutrition in case of contraindications for EN	0	R7	Based on 6 level-2 studies, in critically ill patients with an intact gastrointestinal tract, we recommend that PN not be used routinely, rather early PN should be considered in	R8

	initiated as soon as possible following ICU admission when EN is not feasible						nutritionally high-risk patients with a relative contraindication to early EN.	
30	In patients at either low or high nutritional risk, use of supplemental PN is recommended to be considered after 7-10 days if unable to meet >60% of energy and protein requirements by the EN alone. Initiating supplemental PN prior to this 7-10 day period in critically ill patients receiving some EN does not improve outcomes and may be detrimental to the patient	Moderate	G3	In patients who do not tolerate full-dose EN during the first week in ICU, the safety and benefits of initiating PN should be weighed on a case-by-case basis	GPP – SC	R20	We strongly recommend early supplemental PN and high IV glucose not be used in unselected critically ill patients (i.e. low risk patients with short ICU stay). In patient's not tolerating adequate EN, data are insufficient to recommend when PN should be initiated. Practitioners will have to weigh the safety and benefits of initiating PN in patients not tolerating EN on case-by-case basis.<br><br>Based on 4 level-2 studies, low-dose PN should be considered in critically ill patients who are not malnourished, are tolerating some EN, or when PN is indicated for short-term use (<10 days). Data are insufficient to recommend the use of low-dose PN in the following patients: those requiring long-term (> 10 days) PN; obese and/or malnourished critically ill patients. Practitioners must weigh the safety and benefits	R7.2<br><br>R10.1

							of low-dose PN on a case-by-case basis in these latter patient populations.	
**H) WHEN INDICATED, MAXIMISE THE EFFICACY OF PARENTAL NUTRITION**								
31	Protocols and nutritional support teams are suggested to be used to incorporate strategies to maximise efficacy and reduce associated risk of PN	EC	H1	To enable substrate metabolism, micronutrients (i. e. trace elements and vitamins) should be provided daily with PN	B	R34		
32	Hypocaloric PN dosing (≤20 kcal/kg/d or 80% estimated energy needs) with adequate protein (≥1.2 g protein/kg/d) is suggested to be considered in appropriate patients (high risk or severely malnourished) requiring PN initially over the first week of ICU stay	Low	H2					
33	Withholding or limiting SO-based IVFE during the first week after initiation of PN in critically ill patients at most 100g/wk (often divided into 2 doses/wk) is suggested if there is concern for essential fatty acid deficiency; alternative IVFEs may provide outcome benefit over soy-based IVEFs – no	Very low	H3a, H3b	Administration of intravenous lipid emulsions should be generally a part of PN<br><br>Intravenous lipids (including non-nutritional lipid sources) should not exceed 1.5 g lipids/kg/day and should be adapted to individual tolerance<br><br>Parenteral lipid emulsions enriched with EPA + DHA (Fish oil dose 0.1-0.2 g/kg/d)	GPP – SC<br><br>GPP – SC<br><br>0	R24<br><br>R25<br><br>R33	IV lipids that reduce the load of omega-6 fatty acids/soybean oil emulsions should be considered when PN with intravenous lipids is indicated. However, data are insufficient data to a recommend the type of lipids to be used that reduce the omega-6 fatty acid/soybean oil load in critically ill patients receiving PN	R9.2

	recommendation possible at this time			can be provided in patients receiving PN			Based on 2 level-2 studies, in critically ill patients who are not malnourished, are tolerating some EN, or when PN is indicated for short term use (<10 days), withholding lipids high in SO should be considered. Data are insufficient to recommend withholding lipids high in SO in malnourished critically ill patients or those requiring long-term PN (>10 days). Practitioners must weigh the safety and benefits of withholding lipids high in SO on a case-by-case basis in these latter populations.	R10.2
34	Use of standardized commercially available PN vs. compounded PN admixtures in the ICU has no advantage in terms of clinical outcomes	EC	H4				Data are insufficient to recommend on the use of lipids in total nutrient admixtures (TNA) vs. piggyback in critically ill patients	R10.3
35	A target blood glucose range of 140 or 150-180 mg/dL for the general ICU population is recommended; ranges for specific patient populations (postcardiovascular surgery, head trauma) may differ and are beyond	Moderate	H5				Based on 26 level-2 studies, we recommend avoiding hyperglycaemia (blood sugars > 0 mmol/l) in all critically ill patients. We recommend a blood glucose target of around 8.0 mmol/l. (or 7-9 mmol/l), rather than a more stringent (4.4 to 6.1 mmol/l) or more liberal target range (10 to 11.1 mmol/L). Data are insufficient	R10.4a

	the scope of the ASPEN guideline						to recommend administration of insulin via subcutaneous over IV.	
36	Parenteral glutamine supplementation is recommended not to be used routinely in the critical care setting	Moderate	H6	Parenteral GLN-dipeptide shall not be administered In unstable and complex ICU patients, particularly in those suffering from liver and renal failure	A	R29	Based on 31 studies (10 level-1 studies and 21 level-2 studies), when PN is prescribed to critically ill patients, we recommend parenteral supplementation with glutamine NOT be used. Data are insufficient to recommend the use of intravenous glutamine in critically ill patients receiving EN, but given the safety concerns we also recommend intravenous glutamine not be used in enterally fed critically ill patients.	R9.4a
37	As tolerance to EN improves, the amount of PN energy should be reduced and finally discontinued when the patient is receiving >60% of target energy requirements form EN	EC	H7					
				**I) REFEEDING**				
				In patients with refeeding hypophosphataemia (<0.65 mmol/l or a drop of >0.16 mmol/l), electrolytes should be measured 2-3 times a day and supplemented if needed	GPP – SC	R56		

				In patients with refeeding hypophosphataemia, energy supply should be restricted for 48 h and then gradually increased	B	R57		
				**J) PULMONARY FAILURE**				
38	Specialty high-fat/low-carbohydrate formulations designed to manipulate the respiratory quotient and reduce CO_2 production are suggested not to be used in ICU patients with acute respiratory failure (not to be confused with recommendation E3)	Very low	I1					
39	Fluid-restricted energy-dense EN formulations are suggested to be considered for patients with acute respiratory failure (especially if in a state of volume overload)	EC	I2					
40	Serum phosphate concentrations should be monitored closely and phosphate replaced appropriately when needed	EC	I3					
				**K) RENAL FAILURE**				
41	ICU patients with ARF or AKI are suggested to be placed on a standard enteral formulation and	EC	J1					

	standard ICU recommendations for protein (1.2-2 g/kg ABW/d) and energy (25-30 kcal/kg/d) provision should be followed. If significant electrolyte abnormalities develop, a specialty formulation designed for renal failure (with appropriate electrolyte profile) may be considered							
42	Patients receiving frequent hemodialysis or CRRT are recommended to receive increased protein (maximum ≤2.5 g/kg/d). Protein should not be restricted in patients with renal insufficiency as a means to avoid or delay initiating dialysis therapy	Very low	J2					
**L) HEPATIC FAILURE**								
43	A dry or usual weight is suggested to be used instead of actual weight in predictive equations to determine energy and protein in patients with cirrhosis and hepatic failure, due to complications of ascites, intravascular volume depletion, edema, portal hypertension, and	EC	K1					

	hypoalbuminaemia; nutritional regimens are suggested to avoid restricting proteins in patients with liver failure, using the same recommendations as for other critically ill patients							
44	EN are suggested to be used preferentially when providing nutrition therapy in ICU patients with acute and/or chronic liver disease	EC	K2					
45	Standard enteral formulations are suggested to be used in ICU patients with acute and chronic liver disease. There is no evidence of further benefit of BCAA formulations on coma grade in ICU patients with encephalopathy already receiving first-line therapy with luminal-acting antibiotics and lactulose	EC	K3					
**M) ACUTE PANCREATITIS**								
46	The initial nutritional assessment in acute pancreatitis is suggested to evaluate disease severity to direct nutritional therapy; since disease severity may change	EC, very low	L1a, L1b, L1c					

	quickly, we suggest frequent reassessment of feeding tolerance and need for specialized nutrition therapy; specialized nutrition therapy is suggested not to be provided to patients with mild acute pancreatitis, instead advancing to an oral diet as tolerated. If an unexpected complication or failure to advance to oral diet develops within 7 days, then specialized nutrition therapy should be considered. Patients with moderate to severe acute pancreatitis should have a naso-/oroenteric tube placed and EN started at a trophic rate and advanced to goal, as fluid volume resuscitation is completed (within 24-48 h of admission)							
47	Standard polymeric formula is suggested to be used to initiate EN in patients with severe acute pancreatitis. Although promising, data are currently insufficient to recommend placing a	Very low	L2					

	patient with severe acute pancreatitis on an immune-enhancing formulation at this time							
48	EN is suggested to be used over PN in patients with severe acute pancreatitis who require nutrition therapy. EN is suggested to be provided to patients with severe acute pancreatitis, either by the gastric or jejunal route, as there is no difference in tolerance or clinical outcomes between these 2 levels of infusion	Low	L3a, L3b					
49	In patients with moderate to severe acute pancreatitis who have intolerance to EN, measures should be taken to improve tolerance	EC	L4					
50	Use of probiotics are suggested to be considered in patients with severe acute pancreatitis who are receiving early EN	Low	L5					
51	For patients with severe pancreatitis, when EN is not feasible, use of PN should be considered after 1 week from the onset of the pancreatitis episode	EC	L6					

**N) SURGICAL SUBGROUPS**								
52	Similar to other critically ill patients, early EN with a high protein polymeric diet is suggested to be initiated in the immediate post trauma period (within 24-48 h of injury) once the patient is haemodynamically stable. Immune-modulating formulations containing arginine and FO are suggested to be considered in patients with severe trauma	Very low	M1a, M1b	In critically ill trauma patients, additional EN doses of GLN (0.2-0.3 g/kg/d) can be administered for the first five days with EN; in case of complicated wound healing it can be administered for a longer period of 10-15 d	0	R27		
53	Similar to other critically ill patients, early EN is recommended to be initiated during the immediate post trauma period (within 24-48 h of injury) once the patient is haemodynamically stable. Either arginine-containing immune-modulating formulations or EPA/DHA supplement with standard enteral formula is suggested to be used in patients with TBI	Very low, EC	M2a, M2b	Trauma patients should preferentially receive early EN instead of early PN	B	R50		
54	Early EN (24-48 hours post injury) is suggested to be initiated in patients treated with an OA in the	EC	M3a, M3b					

	absence of bowel injury; an additional 15-30 g/l protein exudate lost for patients with OA is suggested to be provided; energy needs should be determined as for other ICU patients							
				In patients with abdominal or oesophageal surgery, early EN can be preferred over delayed EN	0	R45		
				In critically ill patients with surgical complications after abdominal and esophageal surgery and unable to eat orally, EN (rather than PN) should be preferred unless discontinuity or obstruction of GI tract, or abdominal compartment syndrome is present	GPP – SC	R46		
				In the case of an unrepaired anastomotic leak, internal or external fistula, a feeding access distal to the defect should be aimed for to administer EN	GPP – SC	R47		
				In the case of an unrepaired anastomotic leak, internal or external fistula, or if distal feeding access is not achieved, EN should be withheld and PN may be commenced	GPP – SC	R48		

				In case of high output stoma or fistula, the appropriateness of chyme reinfusion or enteroclysis should be evaluated and performed if adequate	GPP – SC	R49		
55	EN should be provided to burn patients whose GI tracts are functional and for whom volitional intake is inadequate to meet estimated energy needs. PN should be reserved for burn patients for whom EN is not feasible or not tolerated. IC is suggested to be used when available to assess energy needs in burn patients with weekly repeated measures. Burn patients should receive protein in the range of 1.5-2 g/kg/d. EN should be initiated very early (if possible, within 4-6 hours of injury) in a patient with burn injury	EC	M4a, M4b, M4c, M4d	In patients with burns >20% body surface area, additional enteral doses of GLN (0.3-0.5 g/kg/day) should be administered for 10-15 days as soon as EN is commenced	B	R26	Data are insufficient to recommend the use of ornithine ketoglutarate for burn patients and other critically ill patients.	R4.1d
**O) SEPSIS**								
56	Critically ill patients are suggested to receive EN therapy within 24-48 h of diagnosis of severe sepsis/septic shock as soon as resuscitation is complete and the patient	EC	N1	Early and progressive EN should be used in septic patients after haemodynamic stabilization	GPP – SC	R44		

	is haemodynamically stable							
57	Exclusive PN or supplemental PN are not suggested to be used in conjunction with EN early in the acute phase of severe sepsis or septic shock, regardless of patients' degree of nutrition risk	Very low	N2					
58	Due to conflicting studies, no recommendation is possible regarding selenium, zinc, and antioxidant supplementation in sepsis at this time	Moderate	N3				Data are insufficient to recommend IV/PN zinc supplementation in critically ill patients.<br><br>Based on 6 level-1 and 14 level-2 studies, we do not recommend the use of IV/PN selenium supplementation, alone or in combination with other antioxidants, in critically ill patients.	R9.3<br><br>R11.2
59	Trophic feeding (defined as 10-20 kcal/h or ≤500 kcal/d) for the initial phase of sepsis is suggested to be provided, advancing as tolerated after 24-48 h to >80% of target energy goal over the first week. Delivery of 1.2-2 g protein/kg/d is suggested	EC	N4					
60	Immune-modulating formulas are suggested	Moderate	N5					

	not to be used routinely in patients with severe sepsis							
**P) POSTOPERATIVE MAJOR SURGERY (SICU ADMISSION EXPECTED)**								
61	Determination of nutritional risk (e.g. NRS 2002 or NUTRIC score) is suggested to be performed on all postoperative patients in the ICU, and traditional visceral protein levels (serum albumin, prealbumin, and transferrin concentrations) should not be used as markers of nutritional states	EC	O1					
62	EN is suggested to be provided when feasible in the postoperative period within 24 h of surgery, as it results in better outcomes than PN or STD	Very low	O2					
63	Immune-modulating formula (containing both arginine and FO) is suggested to be routinely used in the SICU for the postoperative patient who requires EN therapy	Moderate to low	O3					
64	Enteral feeding is suggested for many patients in difficult postoperative situations	Low to very low	O4					

	such as prolonged ileus, intestinal anastomosis, OA, and need of vasopressors for haemodynamic support. Each case should be individualized based on perceived safety and clinical judgment							
65	For patients who underwent major upper GI surgery and EN is not feasible, PN is suggested to be initiated (only if the duration of therapy is anticipated to be ≥7 days). Unless the patient is at high nutritional risk, PN should not be started in the immediate postoperative period but should be delayed for 5-7 days	EC	O5					
66	Upon advancing the diet postoperatively, patients are suggested to be allowed solid food as tolerated; clear liquids are not required as the first meal	EC	O6					
**Q) CHRONICALLY CRITICALLY ILL**								
67	Chronically critically ill patients (defined as those with persistent organ dysfunction requiring ICU	EC	P1					

	LOS >21 days) are suggested to be managed with aggressive high-protein EN therapy and, when feasible, a resistance exercise program is suggested to be used							
**R) OBESITY IN CRITICAL ILLNESS**								
68	Early EN is suggested to be started within 24-48 h of ICU admission for obese patients who cannot sustain volitional intake	EC	Q1	An iso-caloric high protein diet can be administered to obese patients, preferentially guided by IC measurements and urinary nitrogen losses	0	R51		
69	Nutrition assessment of obese ICU patients is suggested to focus on biomarkers of metabolic syndrome, evaluation of comorbidities, and determination of level of inflammation, in addition to those parameters described for all ICU patients	EC	Q2	In obese patients, energy intake should be guided by IC. Protein delivery should be guided by urinary nitrogen losses or lean body mass determination (using CT or other tools). If IC is not available, energy intake can be based on “adjusted body weight”; if urinary nitrogen losses or lean body mass determination are not available, protein intake can be 1.3 g/kg “adjusted body weight”/d	GPP – cons.	R52		
70	Nutritional assessment of the obese ICU patient is suggested to focus on evidence of central adiposity, metabolic syndrome, sarcopenia,	EC	Q3					

	BMI >40, SIRS, or other comorbidities that correlate with higher obesity-related risk for cardiovascular disease and mortality							
71	High-protein hypocaloric feeding is suggested to be implemented in the case of obese ICU patients to preserve lean body mass, mobilize adipose stores, and minimize the metabolic complications of overfeeding	EC	Q4					
72	For all classes of obesity, the goal of EN regimen should not exceed 65%-70% of target energy requirements as measured by IC. If IC is unavailable, we suggest using the weight-based equation 11-14 kcal/kg/day ABW for patients with BMI in the range of 30-50 and 22-25 kcal/kg/day IBW for patients with BMI >50. Protein should be provided in a range from 2.0 g/kg/day IBW for patients with BMI of 30-40 and ≤2.5 g/kg/day IBW for patients with BMI ≥40	EC	Q5					

73	If available, an enteral formula with low caloric density and a reduced NPC:N is suggested to be used in the adult obese ICU patient. While an exaggerated immune response in obese patients implicates potential benefit from immune-modulating formulas, lack of outcome data precludes a recommendation at this time	EC	Q6					
74	Additional monitoring is suggested to be initiated to assess worsening hyperglycaemia, hyperlipidaemia, hypercapnia, fluid overload, and hepatic fat accumulation in obese critically ill patients receiving EN	EC	Q7					
75	Obese ICU patients with a history of bariatric surgery are suggested to receive supplemental thiamine prior to initiating dextrose-containing IV fluids or nutritional therapy. In addition, evaluation for and treatment of micronutrient deficiencies	EC	Q8	In critically ill patients with measured low plasma levels (25-hydroxy-vitamin D <12.5 ng/ml, or 50 mmol/l), vitamin D3 can be supplemented<br><br>In critically ill patients with measured low plasma levels (25-hydroxy-vitamin D <12.5 ng/ml, or 50 mmol/l) a high	GPP – C<br><br>0	R36<br><br>R37	Data are insufficient to recommend the use of vitamin D in critically ill patients	R12

	such as calcium, thiamin, vitamin B_{12}, fat-soluble vitamins (A, D, E, K), and folate, along with the trace minerals iron, selenium, zinc, and copper, should be considered			dose of vitamin D3 (500,000 UI) as a single dose can be administered within a week after admission				
**S) NUTRITIONAL THERAPY END-OF-LIFE SITUATIONS**								
76	ANH is not obligatory in cases of futile care or end-of-life situations. The decision to provide ANH should be based on evidence, best practices, clinical experience and judgement, effective communication with the patient, family, and/or authorised surrogate decision maker, and with respect for patient autonomy and dignity	EC	R1					

*) Including main and sub topics, **) Each sub topic counted separately, ***) Headings according to the A.S.P.E.N guidelines; ABW – actual body weight; AKI – acute kidney injury; ALI – acute lung injury; ANH – artificial nutrition and hydration; ARDS – acute respiratory distress syndrome; ARF – acute renal failure; ASPEN – American Society for Parenteral and Enteral Nutrition; BBCA - branched-chain amino acid , BMI – body mass index; CCCS – Canadian Critical Care Society; CCCTG – Canadian Critical Care Trials Group; CHO – carbohydrates; DHA – docosahexaenoic acid; EC – expert consensus; EE – energy expenditure; EN – enteral nutrition; EPA – eicosapentaenoic acid; ESPEN - European Society for Clinical Nutrition and Metabolism; FOs – fish oil supplements; GI – gastrointestinal tract; GLN – glutamine; GPP – good practice points; GPP – SC – GPP strong consensus; GRV – gastric residual volume; IBW – ideal body weight; IC – indirect calorimetry; IVFE – intravenous fat emulsion; LOE – level of evidence; LOS – length of stay; MICU – medical intensive care unit; NPC:N - non-protein calorie-nitrogen ratio; NPO – nil per os; OA – open abdomen; PN – parenteral nutrition; RCT – randomised controlled trial; SICU – surgical intensive care unit; TBI – traumatic brain injury; SO – soybean oil; VCO_2 – carbon dioxide production; VO_2 – oxygen consumption.
